# Changes in vasoactive pathways in congenital diaphragmatic hernia associated pulmonary hypertension explain unresponsiveness to pharmacotherapy

**DOI:** 10.1186/s12931-017-0670-2

**Published:** 2017-11-07

**Authors:** Daphne S. Mous, Marjon J. Buscop-van Kempen, Rene M. H. Wijnen, Dick Tibboel, Robbert J. Rottier

**Affiliations:** 1 Department of Pediatric Surgery, Erasmus Medical Center, Sophia Children’s Hospital, Wytemaweg 80, 3015 CN, PO Box 2040, Rotterdam, The Netherlands; 2 Department of Cell Biology, Erasmus Medical Center, Rotterdam, The Netherlands

**Keywords:** Nitric oxide, Endothelin, Prostacyclin, Development, Lung, Vasculature, Vasodilation

## Abstract

**Background:**

Patients with congenital diaphragmatic hernia (CDH) have structural and functional different pulmonary vessels, leading to pulmonary hypertension. They often fail to respond to standard vasodilator therapy targeting the major vasoactive pathways, causing a high morbidity and mortality. We analyzed whether the expression of crucial members of these vasoactive pathways could explain the lack of responsiveness to therapy in CDH patients.

**Methods:**

The expression of direct targets of current vasodilator therapy in the endothelin and prostacyclin pathway was analyzed in human lung specimens of control and CDH patients.

**Results:**

CDH lungs showed increased expression of both ETA and ETB endothelin receptors and the rate-limiting Endothelin Converting Enzyme (ECE-1), and a decreased expression of the prostaglandin-I_2_ receptor (PTGIR). These data were supported by increased expression of both endothelin receptors and ECE-1, endothelial nitric oxide synthase and PTGIR in the well-established nitrofen-CDH rodent model.

**Conclusions:**

Together, these data demonstrate aberrant expression of targeted receptors in the endothelin and prostacyclin pathway in CDH already early during development. The analysis of this unique patient material may explain why a significant number of patients do not respond to vasodilator therapy. This knowledge could have important implications for the choice of drugs and the design of future clinical trials internationally.

## Background

**Fig. 1:**
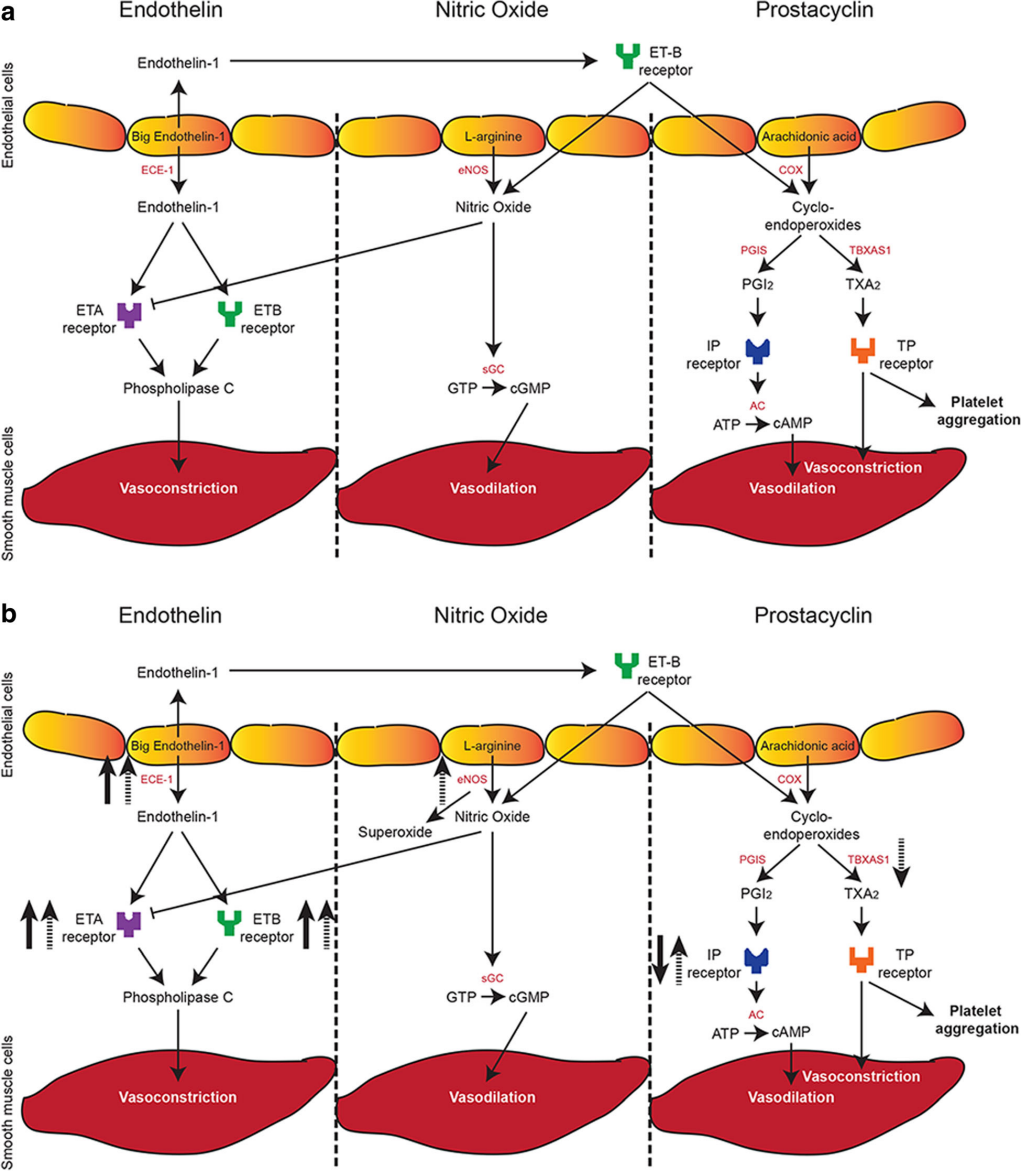
Three major pathways involved in vasodilation and vasoconstriction. **a** Schematic overview of the major pathways and the key proteins involved in vasodilation and vasoconstriction. **b** Aberrant expression of key factors in the three pathways in both human and rat congenital diaphragmatic hernia (CDH). Solid arrows represent up- or downregulation in human CDH, dashed arrows represent up- or downregulation in rat CDH. ECE-1 = endothelin converting enzyme 1, ETA = endothelin A, ETB = endothelin B, eNOS = endothelial nitric oxide synthase, sGC = soluble guanylate cyclase, COX = cyclooxygenase, PGIS = prostaglandin synthase, PGI_2_ = prostaglandin I_2_, AC = adenylate cyclase, TBXAS1 = thromboxane synthase, TXA_2_ = thromboxane

Pulmonary hypertension (PH) is the leading cause of morbidity and mortality in patients with congenital diaphragmatic hernia (CDH) [1]. The altered development of the pulmonary vasculature and the disordered pulmonary vascular remodeling [2] in combination with the imbalance of vasoactive mediators caused by endothelial dysfunction result in the arrest of pulmonary vascular growth in these patients. Current treatment of CDH patients is not evidence based [3] and is derived from studies in adults, leading mainly to off-label and unlicensed use of drugs. Current knowledge is based on compassionate use and case reports, while some patients with CDH were included in trials that were underpowered for definitive conclusions. Even international therapy guidelines are based on consensus only (level 3 evidence) [4]. In 2012, experts evaluated the current antenatal and postnatal management of CDH and emphasized the importance of optimal management of PH in these patients [5]. Worldwide, PH treatment is mainly directed against the receptors of the endothelin (ET) and prostacyclin (PGI_2_) pathways or the conversion of cyclic guanosine monophosphate (cGMP) in the nitric oxide (NO) pathway (Fig. 1a). In spite of these targeted treatments, it is still largely unknown how the different components of these pathways are expressed in lungs of unaffected individuals and CDH patients.

Previous studies reported increased levels of both the endothelin A (ETA) and B (ETB) receptors in human CDH as well as in the nitrofen rat model [6, 7]. Endothelin-1 (ET-1) is a potent vasoconstrictor [8] and is increased in lung tissue of patients with pulmonary hypertension. Moreover, high plasma levels of circulating ET-1 associated with the severity of PH in human CDH [9]. NO reduces the affinity of the ETA receptor for ET-1 and may therefore terminate the ET-1 mediated signaling [10]. NO is synthesized by different NO synthases (NOS): endothelial NOS (eNOS), inducible NOS (iNOS) and neuronal NOS (nNOS), which are all expressed in the lung. However, only eNOS and, to some extent, iNOS, are expressed in the pulmonary vasculature and modulate pulmonary vascular tone [11, 12]. Some human and rat CDH studies showed a decrease in eNOS [13, 14]. However, we and others showed either no differences, or even an increased expression of eNOS in both human and rat CDH [15–19]. PGI_2_ is an important mediator of vasodilation, acting through the prostaglandin-I_2_ receptor (PTGIR) [20]. Several prostacyclin receptor agonists have been used in the treatment of persistent pulmonary hypertension of the newborn with variable effects [21–23]. Limited data are available about the use of these drugs in patients with CDH, but the few available case reports show contrasting results [24–26]. An overview of the current data for human and the rat model is provided (Tables 1 and 2).

**Table 1:** Overview of studies in human CDH

Our group	Others
Increased expression of ETA and ETB (protein level)	- Increased ET-1 (plasma levels and protein level) -[43]
Increased expression of ECE-1 (protein level)	- Increased ET-1 (plasma levels) [9]
	- Increased expression of ETA and ETB (RNA and protein level) [6]
No differences in eNOS expression [16]	- Increased expression of eNOS in arteriolar endothelium and alveolar epithelium (protein level) [17]
	- No differences in eNOS expression (protein level) [19]
	- Decreased expression of eNOS (protein and RNA level) [14]
Decreased expression of Ptgir (protein level)	- No information about prostaglandin I_2_

**Table 2:** Overview of studies in experimental rat CDH

Our group	Others
Increased expression of ETA (RNA and protein level) and ETB (RNA level)	- Increased expression of ETA and ETB (RNA and protein level) [7]
Increased expression of ECE-1 (RNA level)	- Increased expression of ET-1 after 1 and 6 h of ventilation (RNA level) [18]
	- Increased response of arterioles to ET-1 [44]
Increased expression of eNOS (RNA and protein level)	- Increased expression of eNOS (RNA and protein level) [15]
	- Increased expression of eNOS after 1 h of ventilation (RNA level) [18]
	- Decreased expression of eNOS (RNA and protein level) [13]
Increased expression of Ptgir (RNA level) and decreased expression of Tbxas1 (RNA level)	- Increased levels of prostaglandin I_2_ and an increased ratio of prostaglandin I_2_ and thromboxane (protein level) [34]

Since CDH patients respond poorly to current treatment strategies, we hypothesized that these effects might be due to an aberrant expression of important vasoactive factors. Here, we are the first to analyze the expression of the direct targets of the most commonly used vasodilator drugs, as well as some of the important members of the three major vasoactive pathways. Using unique patient lung material, we show an increased expression of both endothelin receptors and the rate-limiting endothelin converting enzyme (ECE-1), as well as a decreased expression of the prostaglandin-I_2_ receptor in human CDH. Moreover, we found changes in the expression of these and other important factors of the pathways in rat CDH (Fig. 1b).

## Methods

### Human lung samples

Human lung samples were retrieved from the archives of the Department of Pathology of the Erasmus Medical Center, Rotterdam. In our high-volume, leading center of the EURO consortium [4], approximately 15 to 20 CDH patients a year are born, which ensures a large experience in the treatment of this disease. Paraffin-embedded lung samples, lacking severe hemorrhage or necrosis, were selected of controls, of CDH patients and of patients with lung hypoplasia or pulmonary hypertension unrelated to CDH. Only lung material of patients with a severe left-sided CDH and a survival of less than 7 h were selected to prevent secondary sequelae. Patient characteristics are described in Table 3.

**Table 3:** Patient characteristics

Disease	GA	Sex	Age of death	Cause of death
Control	18 + 0	Male	–	Abortion
	24 + 6	Female	Minutes	Prematurity
	26 + 5	Female	1 h	Prematurity
	33 + 0	Male	Minutes	Developmental delay
	38 + 3	Male	Minutes	Asphyxia
	38 + 5	Male	1.5 h	Anencephaly
	40 + 0	Female	18 h	Asphyxia
CDH	17 + 6	Male	–	Abortion
	21 + 4	Male	–	Abortion
	36 + 2	Male	Some hours	Respiratory failure
	36 + 2	Female	Some hours	Respiratory failure
	37 + 2	Male	7 h	Respiratory failure
	38 + 0	Male	2 h	Respiratory failure
	40 + 0	Female	Some hours	Respiratory failure
LH	22 + 3	Male	–	Abortion
	28 + 5	Female	15 min	Respiratory failure
	41 + 0	Male	30 min	Respiratory failure
PH	34 + 3	Female	4 days	PPHN
	37 + 1	Male	4 days	Respiratory failure

*GA* gestational age (weeks + days), *CDH* congenital diaphragmatic hernia, *LH* lung hypoplasia, *PH* pulmonary hypertension, *PPHN* persistent pulmonary hypertension of the newborn

### Animal model

The well-established animal model was used, where in short pregnant Sprague-Dawley rats received either 100 mg nitrofen dissolved in 1 ml olive oil or just 1 ml olive oil by gavage on gestational age day E9.5 [27]. Nitrofen induces left-sided CDH in approximately 70% of the offspring, while all pups have pulmonary hypertension. At day E21 pups were delivered by caesarean section and euthanized by lethal injection of pentobarbital.

### Immunohistochemistry staining

Immunohistochemistry (IHC) was performed on 5 μm thick paraffin sections of lungs of both rats and humans according to standard protocols, using the Envision™ detection system (Dako Cytomatic, Glostrup, Denmark) [28]. Briefly, sections were deparaffinized with xylene and rehydrated in gradual series of ethanol, after which antigen retrieval was performed by boiling samples in 10 mM Tris (pH 9.0), 1 mM EDTA. Primary and Horse Redox Peroxidase conjugated secondary antibodies were diluted in antibody dilution buffer (DAKO) with 0,5% Tween-20, and the peroxidase was detected with diamino-benzidine tetrahydrochloride (Fluka, Buchs, Switzerland). Validated primary antibodies used for IHC were Endothelin receptor A (ETA; 1:5000 (rat) 1:100 (human); Alamone, Jerusalem, Israel), Endothelin receptor B (ETB; 1:2500 (rat) 1:500 (human); Alamone), Endothelin Converting Enzyme (ECE-1; 1:500 (human); Abcam, Cambridge, MA, USA), endothelial nitric oxide synthase (eNOS; 1:400 (rat); Thermo Fisher Scientific, Waltham, MA, USA) and prostaglandin-I_2_ receptor (Ptgir; 1:1000 (rat) 1:500 (human); Cayman Chemical, Ann Arbor, Michigan, USA). Negative controls were performed by omitting the primary antibody.

### Quantitative real-time polymerase chain reaction (qPCR)

RNA isolation of whole lungs of rat pups, cDNA synthesis and subsequent qPCR analysis was performed as previously described [28]. The gene-specific primers used are listed in Table 4. *Actb* was used as reference gene.

**Table 4:** Primer sequences

Gene	Sequence (forward 5′- 3′)	Sequence (reverse 5′- 3′)
*Eta*	AACCTGGCAACCATGAACTC	ATGAGGCTTTTGGACTGGTG
*Etb*	CAGGATTCTGAAGCTCACCCTTT	TCCAAAACCAGCAAAAAACTCA
*Et-1*	TGTGCTCACCAAAAAGACAAGAA	GGTACTTTGGGCTCGGAGTTC
*Ece-1*	GCAAGAACATAGCCAGCGAG	CTCCGAGTATCTTCATCCATCC
*eNos*	CATACTTGAGGATGTGGCTG	CCACGTTAATTTCCACTGCT
*Sma*	TGACCCAGATTATGTTTGAGAC	AGAGTCCAGCACAATACCAG
*Ptgis*	CATCAAACAGTTTGTGGTCCT	CAAAGCCATATCTGCTAAGGT
*Ptgir*	CACGAGAGGATGAAGTTTACCA	AATCCTCTGATCGTGAGAGGC
*Tbxas1*	AGACTCAGGTTCCACTTCAG	TCACACCTGCCTTCTATGTC
*Tbxa2r*	ACTGTGAGGTGGAGATGATGG	CAGGATGAAGACCAGCAAGG
*Actb*	AGATGACCCAGATCATGTTTGAG	GTACGACCAGAGGCATACAG

### Statistical analyses

Data are presented as means (SD) for normally distributed variables. Univariate analyses were performed using independent samples t-tests for normally distributed variables. The analyses were performed using SPSS 21.0 for Windows (Armonk, NY, USA: IBM Corp.). All statistical tests were two-sided and used a significance level of 0.05.

## Results

We analyzed the expression of receptors which are targeted during treatment, as well as other critical factors of the different vasoactive pathways, in order to unravel the unresponsiveness of CDH patients to current vasodilator therapies. Therefore, a unique set of lung material of CDH patients was used and the data were verified using the more dynamical nitrofen rat model.

### Human

**Fig. 2:**
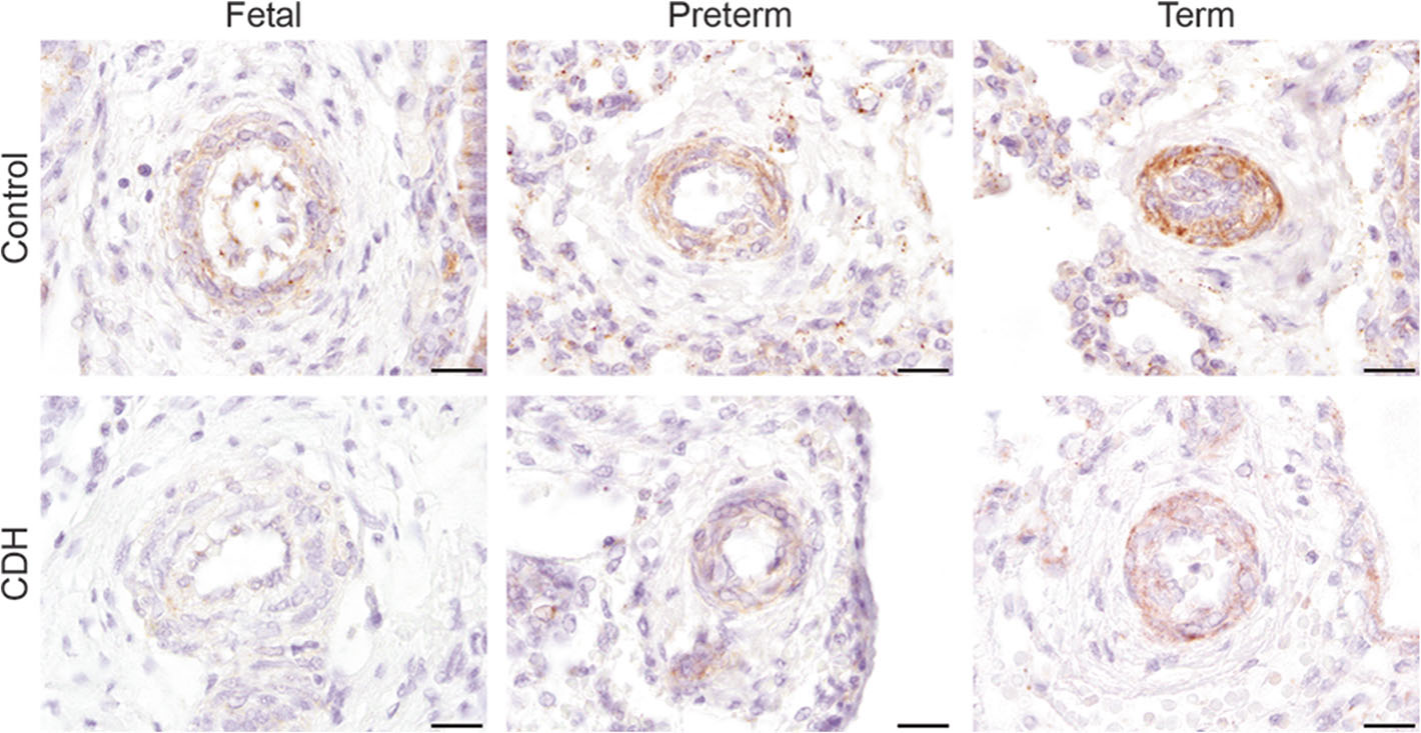
Suppressed progression of prostaglandin-I_2_ receptors during gestation in human CDH. Representative images show progressive expression of PTGIR in the vessels during gestation in human control lungs, which is reduced in lungs of human CDH patients. Scale bars represent 20 μm. Patients: GA 18 + 0, 33 + 0 and 38 + 0 (control), GA 21 + 4, 36 + 2 and 37 + 2 (CDH)

**Fig. 3:**
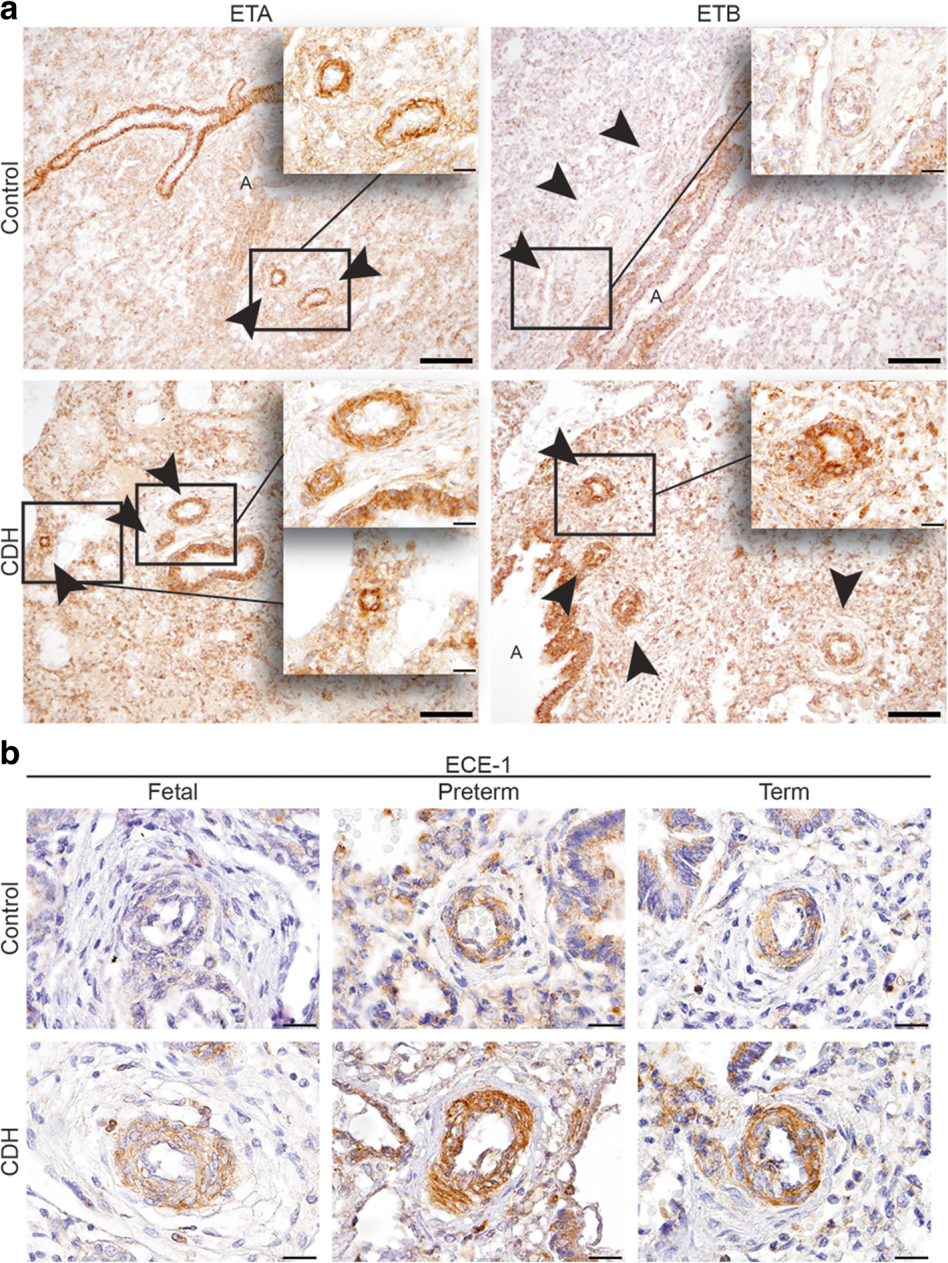
Increased expression of ECE-1 and both ET receptors in human CDH. **a** Representative images show increased expression of ETA in the smaller vessels and clear expression of the ETB receptor in vessels in lungs of CDH patients compared to control. **b** ECE-1 is increasingly expressed in the vessels in human control patients during gestation, whereas the expression is already high in the fetal stage of development in CDH patients. Arrows indicate vessels, A indicates airways. Scale bars represent 100 μm (low power) and 20 μm (high power). Patients: GA 38 + 3 (control), GA 38 + 0 and 37 + 2 (CDH) (A + B). Patients: GA 18 + 0, 26 + 5 and 38 + 0 (control), GA 21 + 4, 36 + 2 and 37 + 2 (CDH) (C + D)

Besides the use of inhaled NO (iNO), current treatment is based on targeting the receptors in both the prostacyclin and endothelin pathway. Therefore, we analyzed the expression of the critical proteins of both pathways in human lung samples of control and CDH patients by immunohistochemistry. Human control lung samples showed little expression of the main target of the prostacyclin therapy, the important prostacyclin receptor PTGIR, in the fetal period. This sharply increased later during gestation at the preterm and term age. However this significant increase was absent in CDH (Fig. 2). The ETA receptor, which induces vasoconstriction and cell proliferation, was expressed in the small (25–50 μm) and larger (>50 μm) vessels as well as in the very small capillaries (<25 μm) in CDH. In contrast, the distribution of the ETA receptor in control lungs was limited to the small and larger vessels only (Arrowheads in Fig. 3a). The ETB receptor, involved in vasodilation through the release of NO and PGI_2_ (Fig. 1), was expressed both in the bronchial epithelium and in some of the larger vessels (>50 μm) in CDH (Arrowheads in Fig. 3a). However, the expression of ETB in control lungs was found only in the bronchial epithelium (Fig. 3a). Next, we analyzed the expression of ECE-1, a membrane-bound metalloprotease that converts big-endothelin into the biologically active compound ET1 and it is a rate-limiting factor in the ET pathway. Early during gestation, in the fetal period, ECE-1 is minimally expressed in the vessels of the human control lung samples, with an increase at preterm and term age. Increased expression of this enzyme at both fetal, preterm and term age was observed in CDH (Fig. 3b), indicating a potential increased bio-availability of active ET-1 already at the fetal stage of development.

**Fig. 4:**
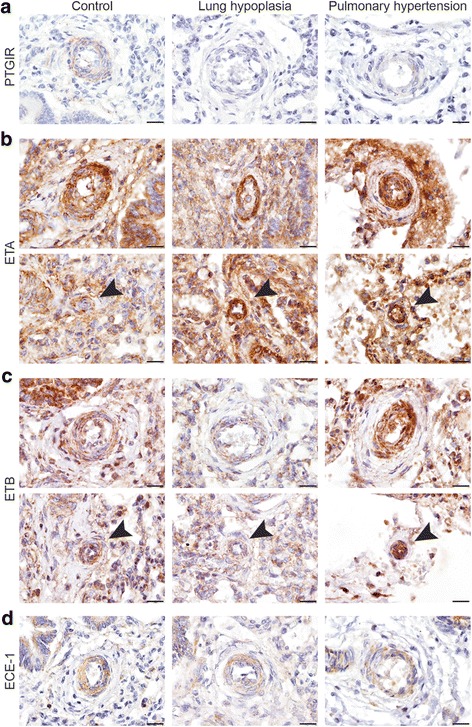
Expression of prostaglandin and endothelin factors in human LH and PH patients. Representative images show expression of PTGIR (**a**), ETA (**b**), ETB (**c**) and ECE-1 (**d**) in patients with lung hypoplasia (LH) or pulmonary hypertension (PH) unrelated to CDH. The expression of ETA is increased in the smaller vessels of patients with LH and PH (**b**), and the expression of ETB is only increased in the vessels of patients with PH (**c**). ECE-1 is not differently expressed in the vessels both LH and PH lung samples (**d**) Scale bars represent 20 μm. Arrows indicate very small vessels. Patients: GA 38 + 3 (control), GA 41 + 0 (LH), GA 34 + 3 (PH)

To exclude that the differences in expression patterns of the crucial prostacyclin – and endothelin receptors and the rate-limiting factor ECE-1 was solely an effect of lung hypoplasia (LH) or PH, we performed immunohistochemistry on lungs of patients with LH and PH with other cause than CDH. The PTGIR receptor expression was reduced in both LH and PH (Fig. 4a). Increased expression of ETA was detected in the smallest vessels in lungs of both LH and PH (Arrowheads in Fig. 4b), whereas increased expression of ETB was only observed in both small and very small vessels of lungs of PH patients (Arrowheads in Fig. 4c). ECE-1 was not expressed differently in both LH and PH lung samples (Fig. 4d).

### Rat

**Fig. 5:**
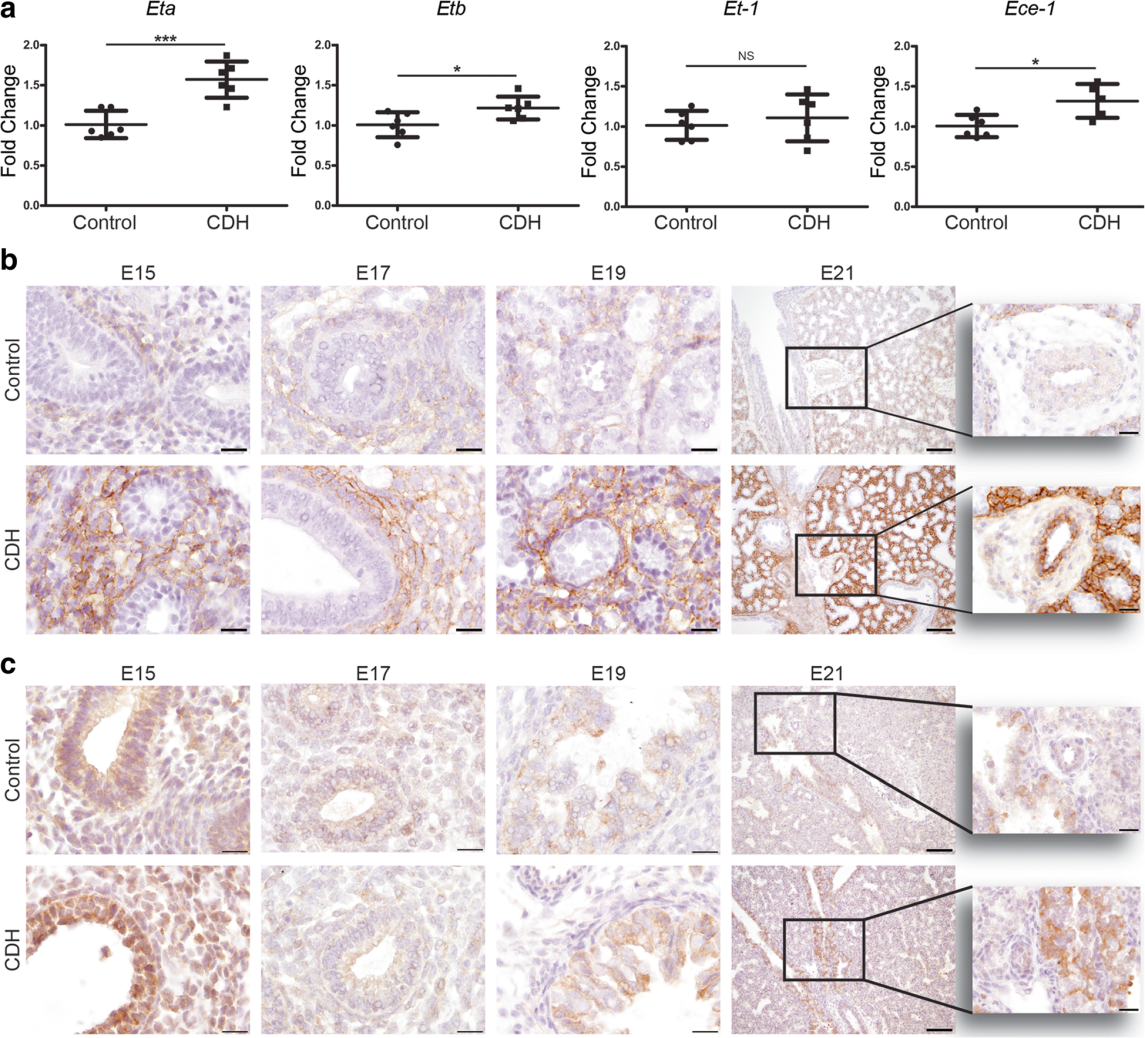
Upregulation of ET-receptors in lungs of rat CDH. **a** Relative expression of the ETA receptor (*Eta*) and ETB receptor (*Etb*) shows a significant increase in rat CDH pups (*p* < 0.001 and *p* < 0.05, respectively), whereas RNA expression of *ET-1* shows no differences between control and CDH and *Ece-1* is significantly increased (*p* < 0.05). **b** Representative images show increased expression of the Eta receptor in the parenchyma of CDH lungs at all stages of development and in the larger vessels at E21. **c** Representative images show expression of the Etb receptor in the bronchial epithelium of both control and CDH lungs at all stages of development. Inserts represent higher magnifications of a pulmonary vessel. *p < 0.05, ***p < 0.001. Error bars represent SD. Arrows indicate vessels, A indicates airways. Scale bars represent 100 μm (low power) and 20 μm (high power)

**Fig. 6:**
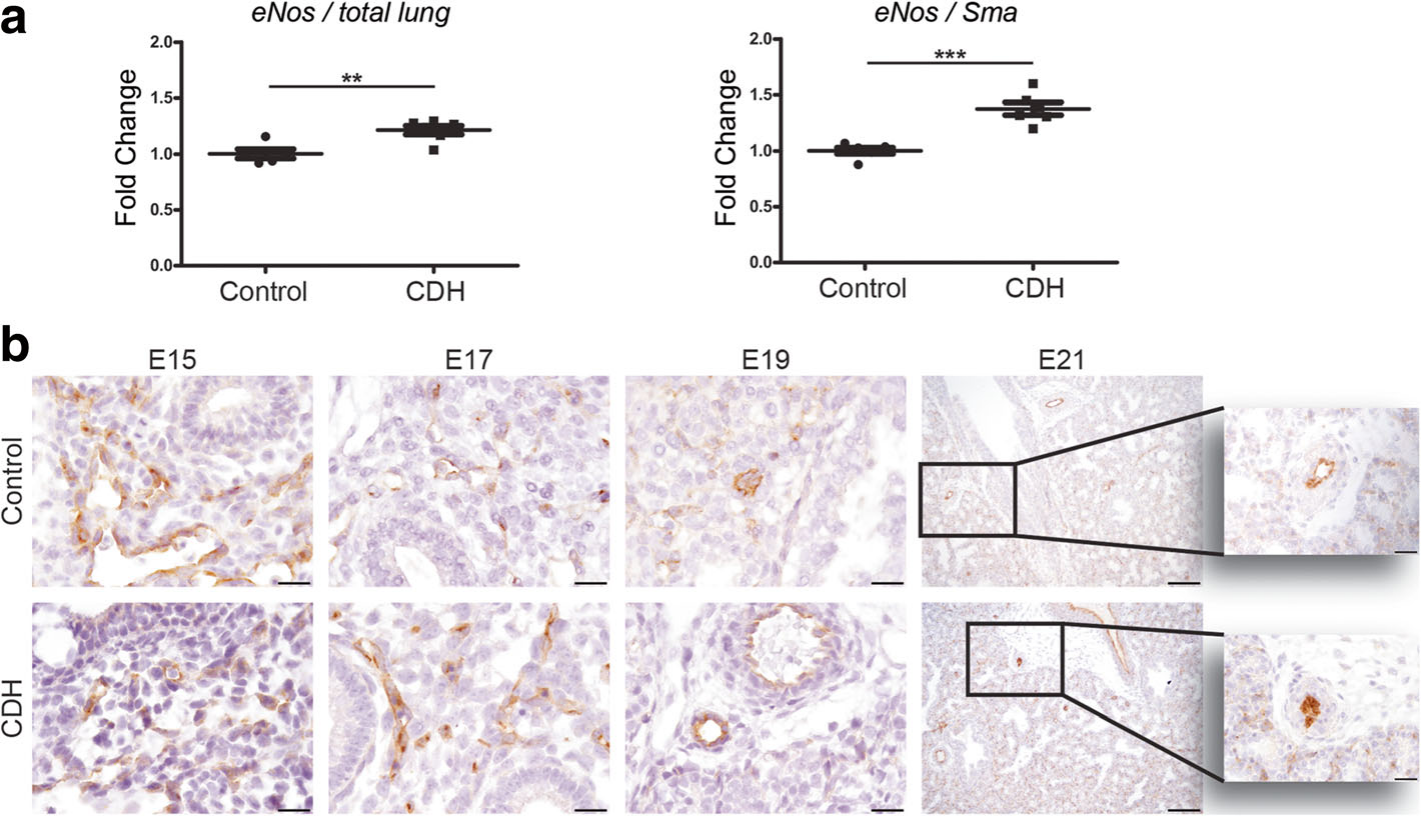
Increased eNOS expression in lungs of CDH rats. **a** *eNOS* expression compared to total rat lungs (left) or to the pulmonary Sma^+^ smooth muscle cells fraction (right) shows increased expression in CDH lungs. **b** Representative images show increased expression of eNOS in the vessels of the lungs of CDH rat pups at E21, but not at other stages of gestation. **p < 0.01, ***p < 0.001. Error bars represent SD. Arrows indicate vessels, A indicates airways. Scale bars represent 100 μm (low power) and 20 μm (high power) and 50 μm

**Fig. 7:**
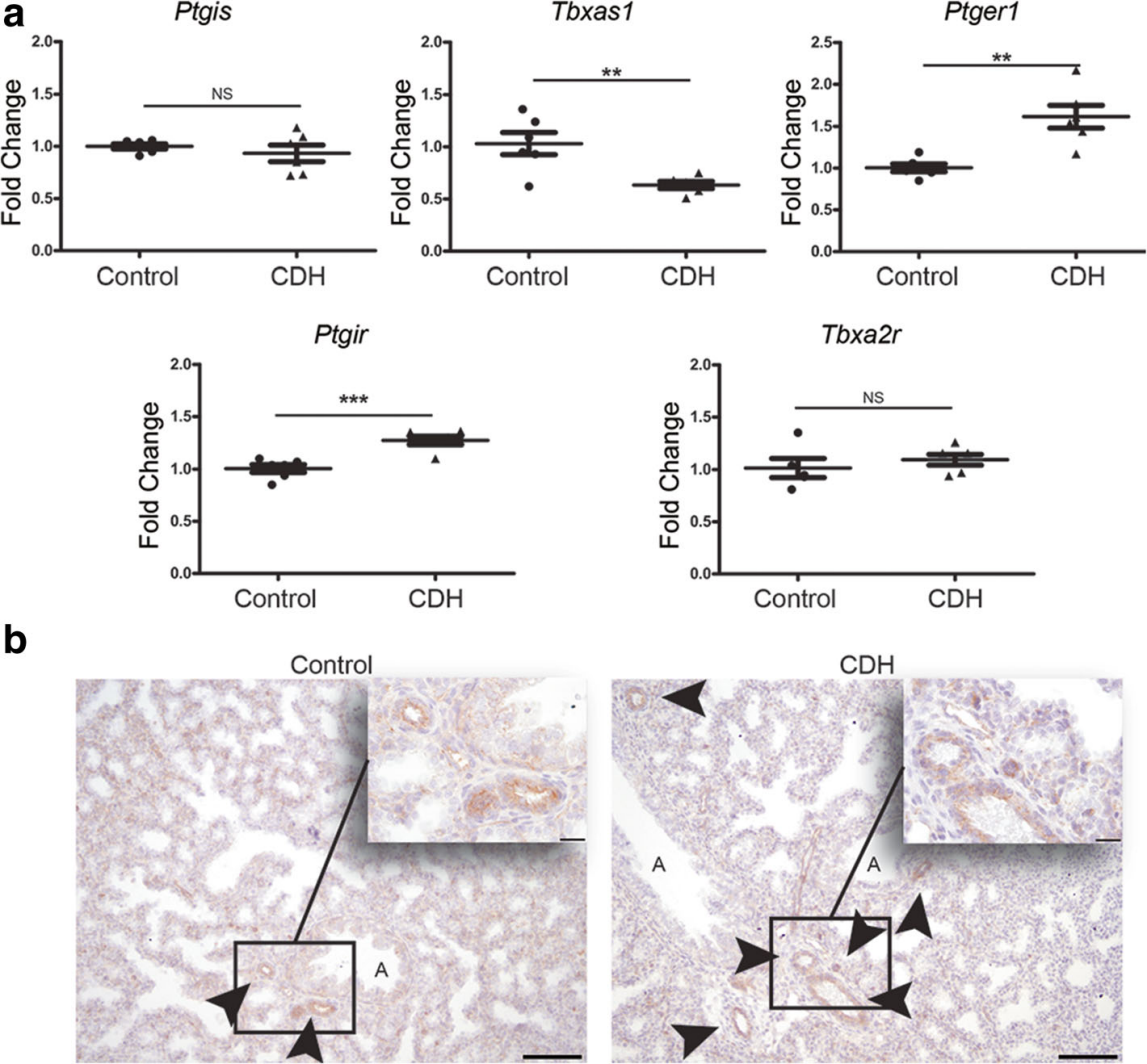
Prostacyclin expression in rat pups. **a** Relative gene expression of prostaglandin I synthase (*Ptgis*), thromboxane synthase (*Tbxas1*), prostaglandin-I_2_ receptor (*Ptgir*) and prostaglandin-E1 receptor (*Ptger1*) in lungs of control and CDH rat pups. **b** Representative images showing the protein expression of PTGIR in the pulmonary vessels in control and CDH lungs.**p < 0.01, ***p < 0.001. Error bars represent SD. Arrows indicate vessels, A indicates airways. Scale bars represent 100 μm (low power) and 20 μm (high power)

In order to validate these interesting human data, we evaluated the expression patterns of the proteins of these three pathways in the nitrofen rat model. This was supplemented with RNA and protein expression analysis of related factors. Real-time qPCR showed that the mRNA expression of both the *Eta* and *Etb* receptors was significantly higher in lungs of E21 pups with CDH compared to those of control pups. We also analyzed the expression of the ETA and ETB ligand, *Et-1*, but no significant differences were found between the groups. However, the mRNA encoding the rate-limiting factor *Ece-1* was significantly increased in CDH compared to control, confirming the human data (Fig. 5a). Next, we analyzed the protein expression pattern of the ET receptors with immunohistochemistry. The ETA receptor was expressed in the small capillaries of both groups at E15 until E21 with a stronger expression level in CDH. At E21 only CDH lungs showed expression of the ETA receptor in the larger vessels (>50 μm) (Fig. 5b). The ETB receptor was expressed in the bronchial epithelium of all lungs without significant differences between control and CDH at all ages (Fig. 5c). There was a significant higher mRNA expression of *eNos* in CDH rats compared to control in relation to all cells as well as in relation to only the smooth muscles cells (Fig. 6a) or endothelial cells (data not shown). This increased expression was clearly detectable with immunostaining in the larger and smaller (<50 μm) vessels at E21. However, no obvious differences were noted earlier during development (E15 till E19) (Fig. 6b). Although there was no difference in expression of prostaglandin-I_2_ synthase (*Ptgis*) between control and CDH rat pups, there was a slight increase in the expression of *Ptgir* and the prostaglandin-E_1_ receptor (*Ptger1*) in CDH at the mRNA level in both the whole lung as well as compared to the number of smooth muscle cells. In contrast, the expression of thromboxane synthase (*Tbxas1*), the enzyme converting prostaglandin H2 into thromboxane A2, which is in turn critical for vasoconstriction, was clearly reduced in CDH, whereas the thromboxane receptor (*Tbxa2r*) did not show significant differences (Fig. 7a). However, immunostaining showed no clear differences in expression of PTGIR in the vessels between both groups (Arrowheads in Fig. 7b).

## Discussion

This is the first combined study showing the aberrant expression of different important factors in the endothelin, NO and PGI_2_ pathways in CDH patients (Fig. 1b) and human patients with LH or PH unrelated to CDH, possibly explaining why a large number of patients do not respond to the current vasodilator therapy. We focused our research on direct targets of the most frequent used drugs to investigate the effectiveness of the current approach and combined this with the analysis of some key factors of the different pathways.

Since our unique human CDH material is scarce and a limiting factor, since only specimens of newborns who lived for a short period were analyzed to prevent secondary morphological changes, supplemental analyses were done on lung tissue of the nitrofen rat model.

In line with previous studies in both human and rat, we found a significant increased expression of the ETA and ETB receptor, important targets of vasodilator therapy, in human CDH patients and the rat model [6, 7, 29]. However, we are the first to show an increased expression of the crucial ECE-1 enzyme in both human pulmonary vessels of CDH patients and whole lung homogenates of nitrofen treated rat pups. ECE-1 converts big ET-1 into the active form of ET-1 and is the rate-limiting step in the production of ET-1 [30]. Although there was no apparent difference in total ET-1 in CDH pups, the higher expression of ECE-1 in lungs of CDH pups may lead to an increase in the active form of ET-1.

Previously, we and others showed no apparent differences in the NO pathway [16, 19]. In contrast to other studies [13, 14, 19, 29], we found an increased expression of eNOS in CDH rats in this study. This may be explained by the decreased NO availability, or by the process of eNOS uncoupling. In case of decreased bioavailability of the cofactor tetrahydrobiopterin (BH4), eNOS produces superoxide instead of NO [31]. This superoxide leads to oxidative stress, which has been observed in vessels of patients with PH [32]. The enhanced activation of the ETA receptor, as mentioned before, might lead to the increase in superoxide production through the induction of reactive oxygen species (ROS) and can thereby induce SMC proliferation and vasoconstriction. Thus, eNOS uncoupling leads to a reduction in NO bioavailability without a necessary change in the amount of eNOS [31]. Furthermore, we have shown a slight increase in expression of the cGMP-specific phosphodiesterase 5 (Pde5) in the NO pathway in nitrofen treated rat pups previously. However, no differences were found in its phosphorylation or its downstream targets, protein kinase G1 (Prkg1) and Prkg2 [27].

The increased expression of PTGIR in control lungs during gestation could result from the gradual increase of placental PGI_2_ toward term [33]. The decreased expression in CDH may be a sign of reduced activation of this pathway. In contrast to our human results, we found no differences in the expression of Ptgir in CDH rat pups and an increase of this receptor on mRNA level. Since PGI_2_ is a potent vasodilator and thromboxane A2 (TXA2) a potent vasoconstrictor, the increased expression of Ptgir and decreased expression of Tbxas1 was unexpected. However, this aberrant balance between PGI_2_ and TXA2 in CDH was already previously described by our group [34]. We showed an increased level of 6-keto-PGF_1α_, the stable metabolite of PGI_2_, and an increased ratio of 6-keto-PGF_1α_ and TXA2 in both lung homogenates and broncho-alveolar lavage (BAL) fluid of nitrofen treated rat pups. The discrepancy between the increased mRNA expression of *Ptgir* in CDH lungs and the absence of differences at the protein level is most likely caused by the difference in sensitivity between qPCRs and IHC. Furthermore, the different results in human and rat could be explained by the fact that we only used the most severe cases of human CDH where the rat model covers all cases.

Current treatment of CDH patients with PH is not evidence based [3] and most patients respond poorly to the used medication. Inhaled NO (iNO) is most commonly used as a first line drug, but its use varies significantly among different centers internationally [35]. In contrast to the promising results of iNO in patients with persistent pulmonary hypertension of the newborn [36], studies in CDH have failed to show its efficacy [35, 37], as no trials have been performed to evaluate the potential role of iNO specifically in CDH patients. Apart from iNO therapy there are some case reports on the use of sildenafil and prostacyclins in CDH patients with variable results [25, 26, 38, 39]. However, administration of enteral sildenafil in neonates leads to highly variable plasma concentrations because of variable gut absorption and/or limited hepatic clearance [40]. The recent availability of intravenous sildenafil may change its application [41], but solid pharmacokinetic data on optimal dosage are still to be published. Treatment with endothelin receptor antagonists is even a bigger problem since these drugs are only available in oral form, while data of its use in newborns are virtually absent concerning dosage absorption and safety. The fact that the current therapy should be considered mainly as” trial and error” and is effective in the minority of patients with CDH strengthens our results that there are possibly more pathways affected. Furthermore, the severity of PH in CDH patients has been known as an important predictor of the outcome and further evaluation of current therapies has been recommended by experts in the field [5]. Future treatment should become more personalized in this group of patients using pathway directed clinical trials and risk stratification [42].

Although the nitrofen rat model is well established, there are still differences in embryonic maturation between rat and human, which may have affected the results. Furthermore, ideally, we would like to be able to directly correlate the findings of aberrant expression of the different vasoactive pathways with the individual response of patients to specific vasoactive drugs. However, given the overall limitations of these types of studies and the lack of material of patients who did respond to one of the three therapies, this remains impossible as repeated lung biopsies would be needed to accomplish this.

## Conclusions

In conclusion, our study shows the aberrant expression of specific vasodilator drug targets and crucial, rate-limiting factors in human CDH and the nitrofen rat model in both the endothelin, NO and PGI_2_ pathway already early during development. Since PH is still a major problem and the most important cause of morbidity and mortality in CDH patients nowadays while current treatment strategies are disappointing, a good insight in these pathways is needed for specific and patient directed targeting of pharmacotherapy.

## Abbreviations

CDH: Congenital diaphragmatic hernia
cGMP: Cyclic guanosine monophosphate
ECE-1: Endothelin converting enzyme 1
eNOS: Endothelial nitric oxide synthase
ET: Endothelin
ET-1: Endothelin 1
ETA: Endothelin receptor A
ETB: Endothelin receptor B
IHC: Immunohistochemistry
LH: Lung hypoplasia
NO: Nitric oxide
PDE5: Phosphodiesterase 5
PGI_2_: Prostacyclin
PH: Pulmonary hypertension
PRKG1: Protein kinase G1
PRKG2: Protein kinase G2
PTGER1: Prostaglandin-E receptor
PTGIR: Prostaglandin-I_2_ receptor
PTGIS: Prostaglandin-I_2_ synthase
qPCR: Quantitative real-time polymerase chain reaction
TBXA2r: Thromboxane receptor
TBXAS1: Thromboxane synthase
TXA2: Thromboxane

## References

[1] Lally KP (2016). Congenital diaphragmatic hernia - the past 25 (or so) years. J Pediatr Surg.

[2] Sluiter I, Reiss I, Kraemer U, Krijger R, Tibboel D, Rottier RJ (2011). Vascular abnormalities in human newborns with pulmonary hypertension. Expert Rev Respir Med.

[3] Puligandla PS, Grabowski J, Austin M, Hedrick H, Renaud E, Arnold M, Williams RF, Graziano K, Dasgupta R, McKee M (2015). Management of congenital diaphragmatic hernia: a systematic review from the APSA outcomes and evidence based practice committee. J Pediatr Surg.

[4] Snoek KG, Reiss IK, Greenough A, Capolupo I, Urlesberger B, Wessel L, Storme L, Deprest J, Schaible T, van Heijst A (2016). Standardized Postnatal Management of Infants with Congenital Diaphragmatic Hernia in Europe: The CDH EURO Consortium Consensus - 2015 Update. Neonatology.

[5] Kotecha S, Barbato A, Bush A, Claus F, Davenport M, Delacourt C, Deprest J, Eber E, Frenckner B, Greenough A (2012). Congenital diaphragmatic hernia. Eur Respir J.

[6] de Lagausie P, de Buys-Roessingh A, Ferkdadji L, Saada J, Aisenfisz S, Martinez-Vinson C, Fund X, Cayuela JM, Peuchmaur M, Mercier JC, Berrebi D (2005). Endothelin receptor expression in human lungs of newborns with congenital diaphragmatic hernia. J Pathol.

[7] Dingemann J, Doi T, Ruttenstock E, Puri P (2010). Upregulation of endothelin receptors a and B in the nitrofen induced hypoplastic lung occurs early in gestation. Pediatr Surg Int.

[8] Davenport AP, Hyndman KA, Dhaun N, Southan C, Kohan DE, Pollock JS, Pollock DM, Webb DJ, Maguire JJ (2016). Endothelin. Pharmacol Rev.

[9] Keller RL, Tacy TA, Hendricks-Munoz K, Xu J, Moon-Grady AJ, Neuhaus J, Moore P, Nobuhara KK, Hawgood S, Fineman JR (2010). Congenital diaphragmatic hernia: endothelin-1, pulmonary hypertension, and disease severity. Am J Respir Crit Care Med.

[10] Michael JR, Markewitz BA (1996). Endothelins and the lung. Am J Respir Crit Care Med.

[11] Fagan KA, Tyler RC, Sato K, Fouty BW, Morris KG, Huang PL, McMurtry IF, Rodman DM (1999). Relative contributions of endothelial, inducible, and neuronal NOS to tone in the murine pulmonary circulation. Am J Phys.

[12] Hoehn T, Stiller B, McPhaden AR, Wadsworth RM (2009). Nitric oxide synthases in infants and children with pulmonary hypertension and congenital heart disease. Respir Res.

[13] North AJ, Moya FR, Mysore MR, Thomas VL, Wells LB, Wu LC, Shaul PW (1995). Pulmonary endothelial nitric oxide synthase gene expression is decreased in a rat model of congenital diaphragmatic hernia. Am J Respir Cell Mol Biol.

[14] Solari V, Piotrowska AP, Puri P (2003). Expression of heme oxygenase-1 and endothelial nitric oxide synthase in the lung of newborns with congenital diaphragmatic hernia and persistent pulmonary hypertension. J Pediatr Surg.

[15] Hofmann A, Gosemann JH, Takahashi T, Friedmacher F, Duess JW, Puri P (2014). Imbalance of caveolin-1 and eNOS expression in the pulmonary vasculature of experimental diaphragmatic hernia. Birth Defects Res B Dev Reprod Toxicol.

[16] de Rooij JD, Hosgor M, Ijzendoorn Y, Rottier R, Groenman FA, Tibboel D, de Krijger RR (2004). Expression of angiogenesis-related factors in lungs of patients with congenital diaphragmatic hernia and pulmonary hypoplasia of other causes. Pediatr Dev Pathol.

[17] Sood BG, Wykes S, Landa M, De Jesus L, Rabah R (2011). Expression of eNOS in the lungs of neonates with pulmonary hypertension. Exp Mol Pathol.

[18] Shinkai T, Shima H, Solari V, Puri P (2005). Expression of vasoactive mediators during mechanical ventilation in nitrofen-induced diaphragmatic hernia in rats. Pediatr Surg Int.

[19] Shehata SM, Sharma HS, Mooi WJ, Tibboel D (2006). Pulmonary hypertension in human newborns with congenital diaphragmatic hernia is associated with decreased vascular expression of nitric-oxide synthase. Cell Biochem Biophys.

[20] Gao Y, Raj JU (2010). Regulation of the pulmonary circulation in the fetus and newborn. Physiol Rev.

[21] De Jaegere AP, van den Anker JN (1998). Endotracheal instillation of prostacyclin in preterm infants with persistent pulmonary hypertension. Eur Respir J.

[22] Kelly LK, Porta NF, Goodman DM, Carroll CL, Steinhorn RH (2002). Inhaled prostacyclin for term infants with persistent pulmonary hypertension refractory to inhaled nitric oxide. J Pediatr.

[23] Sood BG, Delaney-Black V, Aranda JV, Shankaran S (2004). Aerosolized PGE1: a selective pulmonary vasodilator in neonatal hypoxemic respiratory failure results of a phase I/II open label clinical trial. Pediatr Res.

[24] De Luca D, Zecca E, Vento G, De Carolis MP, Romagnoli C (2006). Transient effect of epoprostenol and sildenafil combined with iNO for pulmonary hypertension in congenital diaphragmatic hernia. Paediatr Anaesth.

[25] Olson E, Lusk LA, Fineman JR, Robertson L, Keller RL (2015). Short-term Treprostinil use in infants with congenital diaphragmatic hernia following repair. J Pediatr.

[26] Skarda DE, Yoder BA, Anstadt EE, Lally PA, Greene T, McFadden M, Rollins MD (2015). Epoprostenol does not affect mortality in neonates with congenital diaphragmatic hernia. Eur J Pediatr Surg.

[27] Mous DS, Kool HM, Buscop-van Kempen MJ, Koning AH, Dzyubachyk O, Wijnen RM, Tibboel D, Rottier RJ (2016). Clinically relevant timing of antenatal sildenafil treatment reduces pulmonary vascular remodeling in congenital diaphragmatic hernia. Am J Physiol Lung Cell Mol Physiol.

[28] Rajatapiti P, van der Horst IW, de Rooij JD, Tran MG, Maxwell PH, Tibboel D, Rottier R, de Krijger RR (2008). Expression of hypoxia-inducible factors in normal human lung development. Pediatr Dev Pathol.

[29] Makanga M, Maruyama H, Dewachter C, Da Costa AM, Hupkens E, de Medina G, Naeije R, Dewachter L (2015). Prevention of pulmonary hypoplasia and pulmonary vascular remodeling by antenatal simvastatin treatment in nitrofen-induced congenital diaphragmatic hernia. Am J Physiol Lung Cell Mol Physiol.

[30] Kuruppu S, Smith AI (2012). Endothelin converting Enzyme-1 phosphorylation and trafficking. FEBS Lett.

[31] Roe ND, Ren J (2012). Nitric oxide synthase uncoupling: a therapeutic target in cardiovascular diseases. Vasc Pharmacol.

[32] Bowers R, Cool C, Murphy RC, Tuder RM, Hopken MW, Flores SC, Voelkel NF (2004). Oxidative stress in severe pulmonary hypertension. Am J Respir Crit Care Med.

[33] Walsh SW (2004). Eicosanoids in preeclampsia. Prostaglandins Leukot Essent Fatty Acids.

[34] Ijsselstijn H, Zijlstra FJ, Van Dijk JP, De Jongste JC, Tibboel D (1997). Lung eicosanoids in perinatal rats with congenital diaphragmatic hernia. Mediat Inflamm.

[35] Putnam LR, Tsao K, Morini F, Lally PA, Miller CC, Lally KP, Harting MT (2016). Congenital diaphragmatic hernia study G: evaluation of variability in inhaled nitric oxide use and pulmonary hypertension in patients with congenital diaphragmatic hernia. JAMA Pediatr.

[36] Roberts JD, Fineman JR, Morin FC, Shaul PW, Rimar S, Schreiber MD, Polin RA, Zwass MS, Zayek MM, Gross I (1997). Inhaled nitric oxide and persistent pulmonary hypertension of the newborn. The inhaled nitric oxide study group. N Engl J Med.

[37] Inhaled nitric oxide and hypoxic respiratory failure in infants with congenital diaphragmatic hernia (1997). The Neonatal Inhaled Nitric Oxide Study Group (NINOS). Pediatrics.

[38] Bialkowski A, Moenkemeyer F, Patel N (2015). Intravenous sildenafil in the management of pulmonary hypertension associated with congenital diaphragmatic hernia. Eur J Pediatr Surg.

[39] Noori S, Friedlich P, Wong P, Garingo A, Seri I (2007). Cardiovascular effects of sildenafil in neonates and infants with congenital diaphragmatic hernia and pulmonary hypertension. Neonatology.

[40] Ahsman MJ, Witjes BC, Wildschut ED, Sluiter I, Vulto AG, Tibboel D, Mathot RA (2010). Sildenafil exposure in neonates with pulmonary hypertension after administration via a nasogastric tube. Arch Dis Child Fetal Neonatal Ed.

[41] Steinhorn RH, Kinsella JP, Pierce C, Butrous G, Dilleen M, Oakes M, Wessel DL (2009). Intravenous sildenafil in the treatment of neonates with persistent pulmonary hypertension. J Pediatr.

[42] Akinkuotu AC, Cruz SM, Abbas PI, Lee TC, Welty SE, Olutoye OO, Cassady CI, Mehollin-Ray AR, Ruano R, Belfort MA, Cass DL (2016). Risk-stratification of severity for infants with CDH: prenatal versus postnatal predictors of outcome. J Pediatr Surg.

[43] Kobayashi H, Puri P (1994). Plasma endothelin levels in congenital diaphragmatic hernia. J Pediatr Surg.

[44] Coppola CP, Au-Fliegner M, Gosche JR (1998). Endothelin-1 pulmonary vasoconstriction in rats with diaphragmatic hernia. J Surg Res.

